# Emerging Roles of COX7RP and Mitochondrial Oxidative Phosphorylation in Breast Cancer

**DOI:** 10.3389/fcell.2022.717881

**Published:** 2022-02-01

**Authors:** Shuhei Kamada, Toshihiko Takeiwa, Kazuhiro Ikeda, Kuniko Horie, Satoshi Inoue

**Affiliations:** ^1^ Division of Systems Medicine and Gene Therapy, Saitama Medical University, Saitama, Japan; ^2^ Department of Urology, Graduate School of Medicine, Chiba University, Chiba, Japan; ^3^ Department of Systems Aging Science and Medicine, Tokyo Metropolitan Institute of Gerontology, Tokyo, Japan

**Keywords:** mitochondria, metabolism, breast cancer, OxPhos, ERR

## Abstract

Metabolic alterations are critical events in cancers, which often contribute to tumor pathophysiology. While aerobic glycolysis is a known characteristic of cancer-related metabolism, recent studies have shed light on mitochondria-related metabolic pathways in cancer biology, including oxidative phosphorylation (OXPHOS), amino acid and lipid metabolism, nucleic acid metabolism, and redox regulation. Breast cancer is the most common cancer in women; thus, elucidation of breast cancer-related metabolic alteration will help to develop cancer drugs for many patients. We here aim to define the contribution of mitochondrial metabolism to breast cancer biology. The relevance of OXPHOS in breast cancer has been recently defined by the discovery of COX7RP, which promotes mitochondrial respiratory supercomplex assembly and glutamine metabolism: the latter is also shown to promote nucleic acid and fatty acid biosynthesis as well as ROS defense regulation. In this context, the estrogen-related receptor (ERR) family nuclear receptors and collaborating coactivators peroxisome proliferator-activated receptor-γ coactivator-1 (PGC-1) are essential transcriptional regulators for both energy production and cancer-related metabolism. Summarizing recent findings of mitochondrial metabolism in breast cancer, this review will aim to provide a clue for the development of alternative clinical management by modulating the activities of responsible molecules involved in disease-specific metabolic alterations.

## Introduction

Breast cancer is the leading cause of cancer-related deaths in women worldwide (Sung et al., 2021). Most breast cancers express specific hormone receptors (HRs) for estrogen (estrogen receptor α, ERα) and progesterone (progesterone receptor, PR), and some cancers exhibit human epidermal growth factor receptor 2 (HER2)/erb-b2 receptor tyrosine kinase 2 (ERBB2) gene amplification or overexpression. These HR- and HER2-positive cancers can be treated by endocrine and anti-HER2 therapies, respectively; nevertheless, acquired resistance often develops during therapy (Jordan, 2009; Chien, 2020). Many factors have been clarified as key regulators for endocrine resistance, including estrogen receptor, serine/threonine- and tyrosine-protein kinases, cell cycle regulators, recently well-characterized cancer stem-like cells, and tumor microenvironment. Nevertheless, the issue of acquired endocrine resistance remains to be conquered in clinic. Triple-negative breast cancer (TNBC) is a subtype that lacks ERα, PR, and HER-2 expressions, and its standardized treatment remains to be established. While recent therapeutic agents have improved patient prognosis, such as cyclin-dependent kinase 4/6 inhibitors for HR-positive, HER2-negative, advanced breast cancer (Gao et al., 2020) or poly-ADP-ribose polymerase (PARP) and immune check-point inhibitors for TNBC (Vagia et al., 2020), the development of alternative diagnostic and therapeutic options targeting “cancer’s fuel” may provide novel powerful tools to eradicate breast cancer by its metabolic dependencies and vulnerability. Here we discuss mitochondrial contribution to metabolic alterations in breast cancer cells focusing on recent findings regarding OXPHOS and mitochondrial respiratory supercomplexes. Mitochondrial respiratory supercomplexes are super molecular complexes formed of the assembly of respiratory complexes I, III, and IV, and have a role on efficient energy production. Notably, COX7RP is demonstrated to stimulate the assembly of mitochondrial respiratory supercomplexes and associated with breast cancer. To propose the signaling pathway as a therapeutic target for mitochondrial metabolic alteration, we also discuss that the estrogen-related receptor (ERR) family nuclear receptors and collaborating coactivators peroxisome proliferator-activated receptor-γ coactivator-1 (PGC-1) function as regulatory factors for OXPHOS and mitochondrial respiratory function.

## Altered Metabolic Dependencies of Energy Production in Breast Cancer

Cancer cells undergo metabolic changes that allow them to meet the energy demands required for enhanced proliferation and other aspects of malignancy. Many studies have indicated that metabolic pathways including glycolysis, oxidative phosphorylation (OXPHOS), the tricarboxylic acid (TCA) cycle, amino acid and lipid metabolism, and regulation of reactive oxygen species (ROS) are reprogrammed in cancer cells (Sobanski et al., 2021). The best-known metabolic abnormality in cancer cells is aerobic glycolysis, or the Warburg effect, which is described as the increased uptake of glucose and the conversion of glucose to lactate even in the presence of oxygen. Elevated aerobic glycolysis is beneficial for the growth of tumor cells under a hypoxic environment (Fantin et al., 2006) as well as for the synthesis of macromolecules such as nucleic acids (Lunt and Vander Heiden, 2011). The acidic extracellular microenvironment due to lactate production enhances the growth and invasion of cancer cells (Gatenby and Gillies, 2008). Although aerobic glycolysis has been observed in a variety of cancers, most cancer cells use both aerobic glycolysis and mitochondrial OXPHOS to generate ATP molecules. In terms of breast cancer cells, MCF-7 (HR-positive), SKBR3 (HER2-positive), and MDA-MB-231 (TNBC) depend on glycolysis to fulfil up to 25, 50, and 75% of their ATP requirements, respectively (Wu et al., 2016; Louie et al., 2020), suggesting that the dependencies of energy production may differ among breast cancer phenotypes, as well as genotypes. While hypoxic cancer microenvironments usually increase glucose consumption and glycolysis in tumor cells, cancers with unaffected mitochondria exert OXPHOS to efficiently produce ATP (Gwangwa et al., 2018).

ROS production especially during OXPHOS facilitates tumor progression in one aspect, such as by repressing tumor suppressor phosphatase and tensin homolog (PTEN) activity and subsequently enhancing phosphoinositide 3-kinase (PI3K)/protein kinase B (Akt) pathway (Gorrini et al., 2013). Vice versa, the upregulation of ROS promotes genomic instability that results in cell death in another aspect, thus the elimination of ROS is also important for cancer cell survival by increasing ROS scavengers (Hecht et al., 2016). Mitochondria uptake glutamine to convert it to glutamate, and subsequently to TCA intermediate *α*-ketoglutarate (α-KG), a major anaplerotic pathway mediated by mitochondria (Martínez-Reyes and Chandel, 2020). The glutamine metabolism also produces glutathione, which functions as an antioxidant to eliminate ROS. Therefore, the metabolic cross-talk and balance between glycolysis, the TCA cycle, OXPHOS, and ROS production are key determinants for cancer phenotype and biology.

## OXPHOS and Mitochondrial Respiratory Genes Contribute to Breast Cancer Biology

As described above, OXPHOS plays a role in the pathophysiology of cancers including breast cancer (Zu and Guppy, 2004; Vaupel and Mayer, 2012; Ippolito et al., 2016; Ashton et al., 2018; Ikeda et al., 2019; Schöpf, et al., 2020; Takayama et al., 2020; Becherini et al., 2021). In fact, overexpression of mitochondrial OXPHOS-related proteins including cytochrome *c* oxidase subunit 4 (COX4) has been identified in breast cancer cells (Calderón-González et al., 2015). Notably, mitochondrial respiratory complex activity, detected by staining frozen sections of breast cancer tissues, indicated that OXPHOS is upregulated in cancer cells (Whitaker-Menezes et al., 2011). Several reports indicate that in breast cancer, OXPHOS is regulated by multiple mechanisms such as expression/assembly of subunits of mitochondrial respiratory complexes and formation of mitochondrial respiratory supercomplexes (Table 1). Dysfunctional OXPHOS may also be linked to alterations in mitochondrial morphology or fission/fusion. Mitochondrial voltage-dependent anion channel 1 (VDAC1), which is often overexpressed in breast cancers, regulates the expression of enzymes involved in OXPHOS (Arif et al., 2018). Elevated expression of the mitochondrial protein translation (MPT) pathway genes, leading to an increase in the level of the mitochondria-encoded OXPHOS subunit COX2, has been identified in retinoblastoma tumor-suppressor gene (*RB1*)-deficient TNBC cells (Jones et al., 2016). MicroRNA *miR-663* targets the mitochondrial respiratory complex III assembly factor ubiquinol-cytochrome *c* reductase complex assembly factor 2 (UQCC2) transcript and regulates breast cancer cell proliferation (Carden et al., 2017). Under energy stress conditions, breast cancer cells survive due to enhanced respiratory complex assembly and OXPHOS, which is associated with protein kinase A (PKA)-mediated mitochondrial elongation (Li et al., 2017). Tumor necrosis factor receptor-associated protein 1 (TRAP1) regulates mitochondrial aerobic respiration and mitochondrial fusion, thereby triggering tubular networks, which are involved in oncogenesis in MDA-MB-231 and MCF-7 cells (Zhang et al., 2015).

**TABLE 1:** Regulation of mitochondrial respiratory supercomplex and oxidative phosphorylation (OXPHOS) in breast cancer cells.

Key factor	Function	Regulation/mechanism	Cells or tissues	Ref
			Used for the analysis	
COX7RP	Oncogenic	Mitochondrial respiratory supercomplex assembly	MDA-MB-231	Ikeda et al. (2019)
TNF-α	Oncogenic	Decrease of mitochondrial respiratory supercomplex assembly	MCF-7	Shinde et al. (2021)
RB1	Tumor-suppressive	Downregulation of mitochondria encoded OXPHOS subunits, COX2, through decreasing the expression of MPT genes	BT549, HCC 1937, MCF-7, MDA-MB-231	Jones et al. (2016)
miR-663	Tumor-suppressive	Downregulation of the complex III assembly factor, UQCC2	MCF-7, MDA-MB-231	Carden et al. (2017)
PKA	Oncogenic	Mitochondrial elongation under low nutrient conditions and switching from glycolysis to OXPHOS	MCF-7, MDA-MB-231	Li et al. (2017)
TRAP1	Oncogenic	Maintenance of mitochondrial respiration	MCF-7, MDA-MB-231	Zhang et al. (2015)
VDAC1	Oncogenic	Regulation of the expression of the TCA cycle and OXPHOS enzymes	MDA-MB-231	Arif et al. (2018)
SIRT6	Oncogenic	Upregulation of OXPHOS subunit genes, such as *COX5B*, *NDUFB8*, and *UQCRFS1*, and AMPK activity	MCF-7, MDA-MB-231, MMTV-PyMT mammary tumors	Becherini et al. (2021)
ERRα	Oncogenic	Regulation of *IDH1*, *MDH2*, *OGDH* involved in the TCA cycle and *NDUFA1, NDUFB5,* and *COX8A* in mitochondrial respiratory chain	BT474, MCF-7, SKBR3	Deblois et al. (2009)
				Vernier et al. (2020b)
ERRγ	Oncogenic	Regulation of some enzymes *IDH3A*, *OGDH*, *SUCLG1* involved in the TCA cycle and *NDUFAF4*, *NDUFB5*, *COX8A* in mitochondrial respiratory chain	BT474, MDA-MB-231, SKBR3	Tiraby et al. (2011)
				Vernier et al. (2020b)

AMPK, AMP-activated protein kinase; *ATP5F1B*, *ATP synthase F1 subunit beta*; COX2, cytochrome c oxidase subunit 2; *COX5B*, *cytochrome c oxidase subunit 5B*; COX7RP, cytochrome c oxigenase subunit 7A-related protein; *COX8A*, *cytochrome c oxidase subunit 8A*; CypD, cyclophilin D; ERRα, estrogen-related receptor α; ERRγ, estrogen-related receptor γ; *IDH1*, *isocitrate dehydrogenase 1*; *IDH3A*, *isocitrate dehydrogenase 3A*; *MDH2*, *malate dehydrogenase 2*; MPT, mitochondrial protein translation; *NDUFA1*, *NADH:ubiquinone oxidoreductase subunit A1*; *NDUFAF4*, *NADH:ubiquinone oxidoreductase complex assembly factor 4*; *NDUFB5*, *NADH:ubiquinone oxidoreductase subunit B5*; *NDUFB8*, *NADH:ubiquinone oxidoreductase subunit B8*; *OGDH*, *oxoglutarate dehydrogenase*; PKA, protein kinase A; RB1, Retinoblastoma 1; SIRT6, sirtuin 6; *SUCLG1*, *succinate-CoA ligase GDP/ADP-forming subunit alpha*; TNBC, triple negative breast cancer; TNF-α, tumor necrosis factor α; TRAP1, tumor necrosis factor (TNF) receptor associated protein 1; UQCC2, ubiquinol-cytochrome c reductase complex assembly factor 2; *UQCRFS1*, *ubiquinol-cytochrome c reductase, Rieske iron-sulfur polypeptide 1*; VDAC1, voltage-dependent anion channel 1.

Mitochondrial respiratory complexes (i.e., complexes I, III, and IV) form macromolecular assemblies called supercomplexes in the inner mitochondrial membrane. Our group and others have recently revealed that cytochrome *c* oxidase subunit 7a-related polypeptide (COX7RP)/COX7A2L/SCAF1, which was originally identified as an estrogen-responsive gene, stimulates the mitochondrial respiratory supercomplex assembly (Ikeda et al., 2013; Lapuente-Brun et al., 2013; Williams et al., 2016). Although the precise role of the supercomplex has not been elucidated, the supercomplex formation is assumed to facilitate full activity of the mitochondrial respiratory chain and efficient ATP production in MCF-7 cells (Ikeda et al., 2019). COX7RP overexpression is found in breast cancers and shows a correlation with poor survival of patients with breast cancer. In MCF-7 cells, estrogen-induced COX7RP mediates upregulation of mitochondrial respiration and ATP production, leading to estrogen-dependent cell growth. Moreover, COX7RP overexpression contributes to a hypoxia tolerance phenotype in MCF-7 cells through increasing respiratory supercomplex assembly and oxygen consumption, and decreasing ROS levels even in hypoxia. Metabolomic analysis reveals that COX7RP modulates the steady-state levels of TCA cycle intermediates, including higher levels of oncometabolites such as fumaric acid and succinic acid in hypoxia (Yang et al., 2013). This metabolic alteration can be caused by upregulated production of succinic acid and malic acid from glutamine, suggesting partial use of the half part of TCA cycle. Glutamine metabolism fuels the TCA cycle, nucleic acid and fatty acid biosynthesis, and redox regulation in cancer progression (Kodama et al., 2020). COX7RP also mediates breast cancer cell proliferation and invasion under thapsigargin treatment, suggesting a role in stress-inducible metabolic regulation (Zhang et al., 2016).

In breast cancer progression, the tumor microenvironment plays a critical role via promoting inflammation that can modulate mitochondrial function and metabolism. In particular, TNF-α is a pro-inflammatory cytokine secreted by tumor-associated macrophages and cancer cells themselves. It is recently reported that TNF-α decreases the amount and activity of mitochondrial respiratory supercomplex containing complex I and complex IV more potently in TNBC MDA-MB-231 cells compared to ER-positive MCF-7 cells, suggesting that TNF-α regulates the growth of relatively aggressive breast cancer cells by modulating formation and function of mitochondrial respiratory supercomplexes (Shinde et al., 2021). It is also possible to speculate that TNF-α effects on the estrogen-responsive breast cancer cells through signaling pathways other than the mitochondrial respiratory supercomplex formation and metabolism. Taking together with our previous report, in which estrogen-responsive COX7RP stimulates mitochondrial respiratory supercomplex assembly and has a critical role on estrogen-responsive MCF-7 cells (Ikeda et al., 2019), estrogen and TNF-α can coordinately regulate metabolic adaptation of breast cancer cells in a cell-context- and a microenvironment-dependent manner. Furthermore, it is also notable that a mitochondrially targeted agent deferoxamine, an inhibitor of iron-sulfur [Fe-S] cluster/heme biogenesis, suppresses tumor growth and metastasis both in MCF-7 and MDA-MB-231 cells thorough a decrease in mitochondrial respiratory supercomplex assembly (Sandoval-Acuña et al., 2021), indicating that the respiratory supercomplex assembly is a promising therapeutic target for breast cancer cells.

Consistent with our findings (Ikeda et al., 2019), a recent report also revealed that COX7RP facilitates the formation of mitochondrial respiratory supercomplexes and retention of respiratory activity in pancreatic cancer cells even in hypoxic condition leading to a phenotype resistant for hypoxia (Hollinshead et al., 2020). In addition, COX7RP overexpression is associated with poor prognosis of patients with hepatocellular carcinoma and COX7RP promotes the growth and metastasis of HCC through the induction of cell cycle progression and epithelial to mesenchymal transition (Wang et al., 2020). These findings suggest that COX7RP can regulate tumorigenesis in various types of cancers.

## ERRS and PGC-1S Are Key Transcriptional Regulators in Energy Production and Related to Breast Cancer

As described in the previous section, mitochondrial OXPHOS and respiratory supercomplex assembly are involved in cancer-associated metabolic alteration and energy production. Therefore, elucidation of regulators for OXPHOS and mitochondrial respiratory supercomplex assembly will provide a possible target(s) for breast cancer therapy as well as clarifying the signaling pathway. While the precise transcriptional regulation of OXPHOS-related genes in breast cancers remains to be elucidated in clinical breast cancer tissues, it will be useful to review the clinical relevance of OXPHOS-related transcriptional factors in breast cancers, particularly that of the estrogen-related receptor (ERR) family nuclear receptors and collaborating coactivators peroxisome proliferator-activated receptor-γ coactivator-1 (PGC-1).

In terms of the metabolic pathways of energy production, ERRs and PGC-1 are essential transcriptional regulators for mitochondrial biogenesis, energy production, and cancer-related metabolism. ERRs are orphan nuclear receptors that have the sequence similarity with ERα but no endogenous ligands and constitutively exhibit transcriptional activity. Instead of an endogenous ligand, PGC-1α and PGC-1β can function as protein ligands for ERRs and play important roles in metabolic reprogramming (Kamei et al., 2003; Skrzypczak et al., 2013; Vernier and Giguère 2021).

ERRs and their protein ligands PGC-1s have clinical relevance in breast cancer. High expression of ERRα (Suzuki et al., 2004) or PGC-1α (LeBleu et al., 2014) was correlated with poor prognosis of patients with breast cancer. Positive correlation of ERRα with ERBB2/HER2 mRNA levels (Ariazi et al., 2002) and amplified in breast cancer-1 (AIB1) protein levels (Heck et al., 2009) were observed in breast tumors. Recently, ERRα is revealed as a poor prognostic factor in patients with TNBC (Ye et al., 2020; Danza et al., 2021). ERRα (Fradet et al., 2011) and PGC-1α (LeBleu et al., 2014; Andrzejewski et al., 2017) are further associated with metastases in breast cancer patients.

ERRγ and its putative target expression is likely associated with worse prognosis in tamoxifen-treated ER-positive and chemotherapy-treated ER-negative breast cancer patients (Heckler et al., 2014; Madhavan et al., 2015). ERRγ may promote tamoxifen resistance, although its role in cell proliferation remains controversial (Riggins et al., 2008; Ijichi et al., 2011; Tiraby et al., 2011; Heckler et al., 2014).

ERRβ is rather assumed as a better prognostic factor as an inverse correlation between the mRNA expression with the prognosis of TNBC patients (Krishna et al., 2018; Fernandez et al., 2020). ERRβ may inhibit ERα activities (Tanida et al., 2015), or attenuate the cell cycle progression (Krishna et al., 2018).

## ERR/PGC-1-MEDIATED Metabolic Reprogramming in Breast Cancer

**FIGURE 1:**
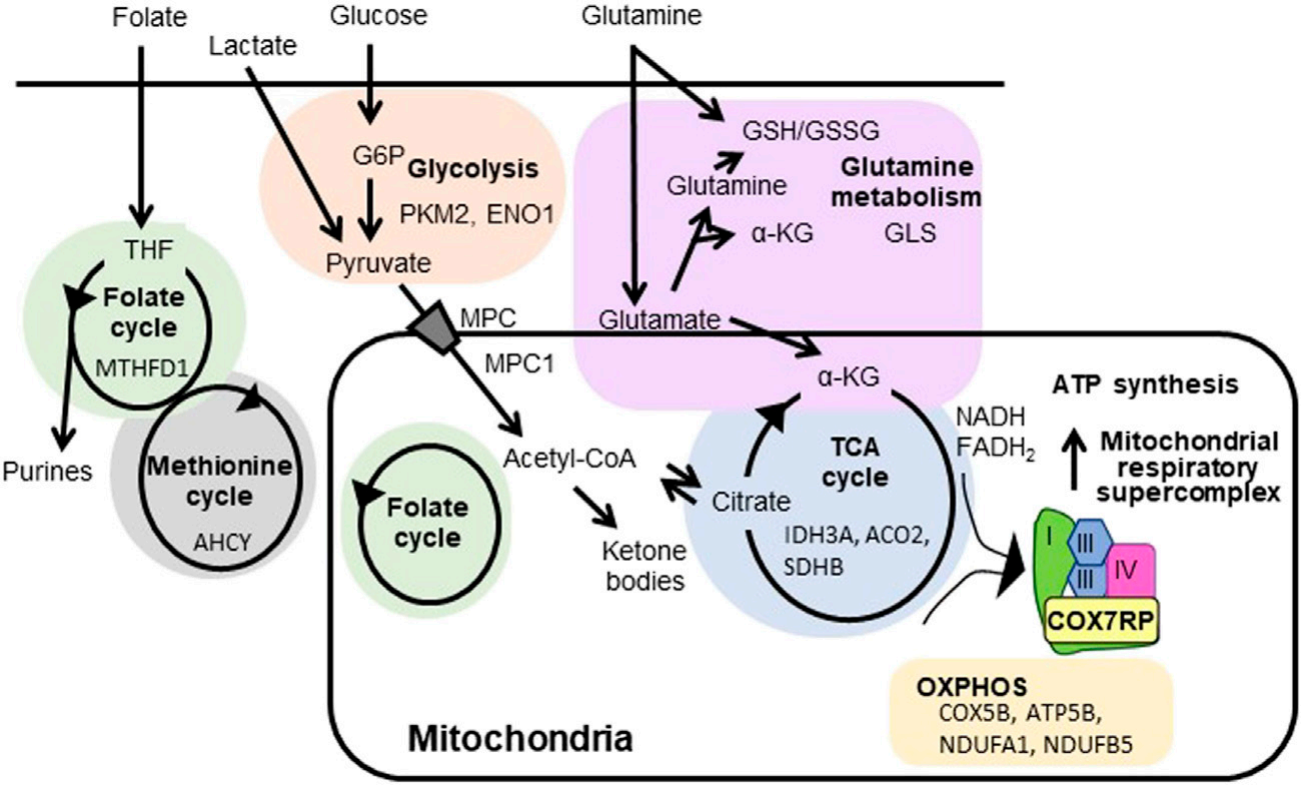
Regulation of mitochondrial respiratory supercomplex assembly by COX7RP and metabolism by ERRs. COX7RP functions as a promoting factor for mitochondrial respiratory supercomplex assembly, leading to efficient ATP production. Metabolic pathways reported to be promoted by ERRs in breast cancer cells are also indicated with the representative target genes. COX7RP, cytochrome *c* oxidase subunit 7a related polypeptide; *α*-KG, *α*-ketoglutarate; G6P, glucose-6-phosphate; GSH, the reduced glutathione; GSSG, glutathione disulfide; MPC, mitochondrial pyruvate carrier protein; THF, tetrahydrofolate; PKM2, pyruvate kinase; ENO1, enolase 1; MTHFD1, methylenetetrahydrofolate dehydrogenase, cyclohydrolase and formyltetrahydrofolate synthetase 1; AHCY, adenosylhomocysteinase; MPC1, mitochondrial pyruvate carrier 1; NDUFA1, ubiquinone oxidoreductase subunit A1; NDUFB5, ubiquinone oxidoreductase subunit B5; COX5B, cytochrome c oxidase subunit 5B; ATP5B, ATP synthase, H+ transporting mitochondrial F1 complex, beta subunit; IDH3A, isocitrate dehydrogenase (NAD(+)) three catalytic subunit alpha; ACO2, aconitase 2; SDHB, succinate dehydrogenase complex flavoprotein subunit B; GLS, glutaminase.

Figure 1 shows schematic diagram of mitochondrial respiratory supercomplex assembly by COX7RP and metabolism by ERRs (Giguère, 2008; Misawa and Inoue, 2015). ERRs regulate the expression of enzymes involved in the TCA cycle as well as the mitochondrial respiratory chain complex subunits to modulate mitochondrial respiration activity (Stein et al., 2008; Deblois et al., 2009; Tiraby et al., 2011; Vernier et al., 2020b). In MCF-7 cells, introduction of a customized PGC-1α that selectively binds to and activates ERRs causes upregulation of IDH3A, a subunit of isocitrate dehydrogenase three which catalyzes isocitrate to *α*-ketoglutarate as part of the TCA cycle (Stein et al., 2008). Genome-wide screening of direct ERRα target genes in breast cancer cells (MCF-7 and SKBr3) identified NDUFA1 and NDUFB5, both of which are subunits of the mitochondrial respiratory complex I (Deblois et al., 2009). The paper also reported that ATP5B, a subunit of mitochondrial ATP synthase, is also regulated by ERRα. Through the investigation of ROS homeostasis in breast cancer BT474 cells, ERRα and ERRγ are revealed to modulate expression of genes in TCA cycle including SDHB and ACO2, and glutamine/glutathione metabolism including GLS (Vernier et al., 2020b). Interestingly, ERRα is implicated in cholesterol-induced metabolic reprogramming in breast cancer cells through the regulation of OXPHOS genes including NDUFB7, ATP5L, and COX5B in MDA-MB-231, MCF-7, and TNBC-PDX cells (Ghanbari et al., 2021).

Because ERRs also exert various functions in a broad range of metabolic pathways, ERR-dependent non-mitochondrial pathways can also be potential therapeutic targets in breast cancer management. For example, ERRα and ERRγ increase the expression of glycolytic genes in cooperation with PGC-1α/β and upregulate glycolysis in MCF-7 and T47D breast cancer cells (Cai et al., 2013). ERRs also interact with hypoxia-inducible factor (HIF)-1/2 to promote transcription of HIF target genes, including glycolytic genes such as pyruvate dehydrogenase kinase 1 (*PDK1*) and phosphoglycerate kinase 1 (*PGK1*) (Ao et al., 2008). In addition, ERRα has been suggested to regulate mitochondrial pyruvate transport. ERRα inhibition downregulates mitochondrial pyruvate carrier 1 (MPC1), which impairs pyruvate transport into the mitochondria (Park et al., 2019). Moreover, ERRα and PGC-1α regulate the expression of genes involved in the folate cycle (Audet-Walsh et al., 2016) and the methionine cycle (Vernier et al., 2020a). Furthermore, ERRα, ERRγ, and PGC-1α regulate the enzymes involved in glutamine metabolism in HER2-positive breast cancer cells (McGuirk et al., 2013; Deblois et al., 2016; Vernier et al., 2020b), which is implicated in resistance to the HER2 inhibitor lapatinib (Li et al., 2020; Vernier et al., 2020b). A recent study showed that ERRα and ERRγ modulate ROS homeostasis, and that ERRγ is associated with resistance to paclitaxel, an anticancer drug that induces ROS. Inhibition of ERRγ by the selective inverse agonist GSK5182 increases sensitivity of organoids generated from TNBC patient-derived xenografts to paclitaxel (Vernier et al., 2020b). Furthermore, ERRα-PGC-1α/β signaling pathway plays an important role in promoting resistance to doxorubicin and epirubicin in MCF-7 cells (McGuirk et al., 2021). Namely, PGC-1α and ERRα are upregulated in doxorubicin- and/or epirubicin-resistant cells generated from MCF-7, leading to the enrichment of these transcription factors at the promoters of genes that contribute to glutathione metabolism, oxidative response, and drug efflux, whereas the knockdown of PGC-1α/β impairs the cell growth and survival.

Regulation of mitochondrial respiration by ERRs affects the stemness of breast cancer cells. Cancer cells that possess self-renewal ability and multi-lineage differentiation are called cancer stem-like cells (CSCs) or tumor-initiating stem-like cells (TICs), which play an essential role in the growth, recurrence, and heterogeneity of tumors (Clegg et al., 2020; van Schie and van Amerongen, 2020). A previous study has shown that treatment with XCT790, an inhibitor of the ERRα-PGC-1α/β signaling pathway, reduces the anoikis resistance of CD44^high^/CD24^low^ MCF-7 cells that represent the CSC sub-population (De Luca et al., 2015). In addition, XCT790 treatment suppresses mammosphere formation by MCF-7 cells, which reflects stem cell activity. XCT790 inhibits mitochondrial respiration, and treatment with the mitochondrial cofactor acetyl-L-carnitine (ALCAR) rescues the decrease in mammosphere formation induced by XCT790, thereby suggesting that mitochondrial respiratory activity is important for the survival and propagation of CSCs.

## Conclusion

This review summarizes the pathophysiological relevance of mitochondrial metabolism in breast cancer. In particular, metabolic dependencies on OXPHOS and TCA cycle are paid attention in tumors including breast cancer, as exemplified by the function of mitochondrial respiratory supercomplex assembly factor COX7RP. In metabolic alterations, transcriptional factors ERRs and their coactivators PGC-1s contribute to breast cancer progression and metastasis by modulating the transcription of their targets including OXPHOS-related genes and oncogenic genes, such as ERBB2 and MYC. As ERRs and PGC-1s are necessary factors in metabolic alterations as well as in early developmental stages and cancer stemness, the inhibition of ERR/PGC-1 pathway efficiently represses CSC proliferation and will be expected to be applied to clinical management for therapy-refractory cancers. Nevertheless, ERRs and PGC-1s are initially essential transcription factors in normal tissues with high energy demands, thus further studies may enable to develop selective inhibitors for ERR/PGC-1 pathway in cancers minimizing side effects on normal tissues.
